# Bridging Therapy and Risk of Bleeding and Thrombosis in Continuous-Flow Left Ventricular Assist Device Patients: A Quasi-Experimental Study

**DOI:** 10.1097/MAT.0000000000002447

**Published:** 2025-04-29

**Authors:** Eleonora Camilleri, Robin P. W. Roovers, Eva Janssen, Jurjen F. Krommenhoek, Frederikus A. Klok, Meindert Palmen, J. Wouter Jukema, Nienke van Rein, Laurens F. Tops

**Affiliations:** From the * Department of Clinical Epidemiology, Leiden University Medical Center, Leiden, the Netherlands; † Department of Clinical Pharmacy & Toxicology, Leiden University Medical Center, Leiden, the Netherlands; ‡ Department of Cardiology, Leiden University Medical Center, Leiden, the Netherlands; § Department of Medicine - Thrombosis and Hemostasis, Leiden University Medical Centre, Leiden, the Netherlands; ¶ Anticoagulation Clinic Leiden, Leiden, the Netherlands; ∥ Department of Cardiothoracic Surgery, Leiden University Medical Center, Leiden, the Netherlands; # Netherlands Heart Institute, Utrecht, the Netherlands.

**Keywords:** ventricular assist device, hemorrhage, heparin, low-molecular-weight, thrombosis

## Abstract

Bridging with low-molecular-weight heparin (LMWH) is recommended in continuous-flow left ventricular assist device (CF-LVAD) patients during subtherapeutic international normalized ratios (INRs). We aimed to assess the risk of adverse events during bridging in patients implanted at Leiden University Medical Center between 2010 and 2024. Incidence rates and hazard ratios of major bleeding, thromboembolic events, neurologic complications, and death with 95% confidence intervals (95% CI) were estimated by time-dependent Cox regression. Using a regression discontinuity design, we mimicked a trial by comparing patients during LMWH treatment due to a subtherapeutic INR to patients during INRs just in target range and no LMWH, considering INRs ±0.1, ±0.2, ±0.3, ±0.4, and ±0.5 around the lower INR target range. Ninety-two patients were included, with a median age of 63 years, 73 (79%) were male and 43 (47%) had Interagency Registry for Mechanically Assisted Circulatory Support (INTERMACS) 3. Major bleeding rates were increased during bridging in all analyses. Bridging had 3.7-fold (95% CI, 1.6–8.7) increased major bleeding risk compared with no bridging considering INRs ±0.1, and 2.8-fold (95% CI, 1.4–5.5) considering INRs ±0.5. Thromboembolic events were infrequent and not different between the two groups. Neurologic complications occurred more frequently during bridging. Moreover, the risk of mortality was 25.0-fold (95% CI, 3.6–173.1) increased during *versus* no bridging. Therefore, bridging should be considered with caution in CF-LVAD patients.

## Graphical abstract

**Figure:**
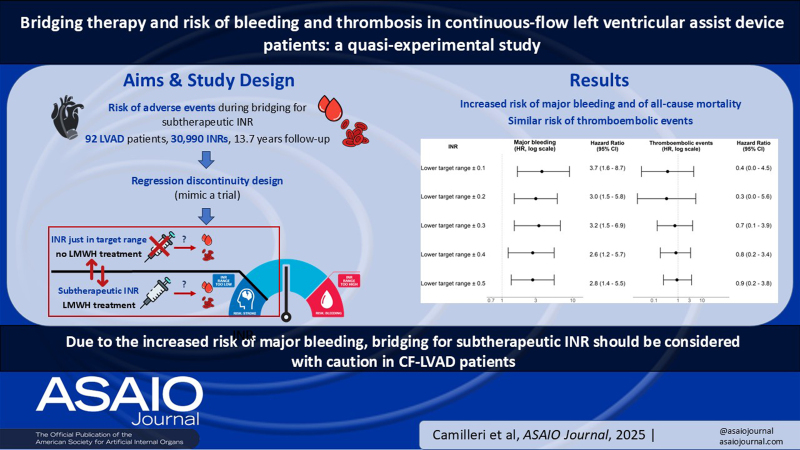

## Background

The survival of end-stage heart failure (HF) patients has greatly improved since the use of continuous-flow left ventricular assist devices (CF-LVADs), as either bridge to heart transplantation or as destination therapy.^1^ In a randomized clinical trial, it was shown that patients with CF-LVAD had a 27% mortality reduction after one year compared with patients receiving medical therapy only.^2^ Moreover, LVAD implantation is associated with overt improvements in quality of life.^3,4^ However, despite obvious advantages, these patients still suffer from numerous device-related adverse events, including bleeding and thromboembolic complications.^4^

In particular, pump thrombosis is a feared complication in CF-LVAD patients (particularly in the former HeartWare device [HVAD]), with an incidence of 0.08 events per patient-year.^5^ In addition, CF-LVAD patients are at increased risk of other thromboembolic complications such as ischemic stroke, accounting for an incidence of 0.19 events per year.^6^ To prevent such complications, adequate antithrombotic therapy is essential. Antithrombotic therapy, however, amplifies the risk of bleeding, which is the most common adverse event in CF-LVAD patients.^7^ In particular, gastrointestinal bleedings are the most prevalent bleeding complication with an incidence of 0.6 events per patient-year within 90 days after implantation.^8^ More than one-third of CF-LVAD patients are rehospitalized at least once in the first month after implantation, due to the occurrence of major bleeding.^9,10^ In addition to antithrombotic therapy, various mechanisms contribute to the increased risk of bleeding, such as acquired qualitative von Willebrand factor deficiency and development of arteriovenous malformation in the gastrointestinal tract.^11^

Antithrombotic therapy for CF-LVAD patients consists of vitamin K antagonists (VKAs) and antiplatelet therapy. In the Netherlands, VKA treatment is managed by anticoagulation clinics who monitor patients closely through international normalized ratio (INR) measurements and required dose adjustments. The INR target range is individualized per patient, based on comorbidities and adverse events.^12^ In CF-LVAD patients, INR is typically monitored regularly (every 2–3 days), to minimize thrombotic and bleeding complications due to off-target anticoagulation. In case of an INR below target (*i.e.*, a subtherapeutic INR), guidelines recommend initiation of bridging therapy to prevent thrombosis.^13,14^ In our center, bridging therapy consists of the addition of a low-molecular-weight heparin (LMWH). However, this recommendation is only based on few studies with small populations and without the consensus of experts.^13,14^

In fact, no randomized clinical trial has evaluated whether the addition of LMWH during a period of subtherapeutic anticoagulation reduces the risk of thromboembolic events without increasing major bleeding in CF-LVAD patients who are already treated with double antithrombotic therapy. Only a few observational studies have been conducted, some suggesting that bridging therapy with LMWH increases the risk of major bleeding,^7,15^ yet not all.^16,17^ However, as patients are not randomly assigned to LMWH treatment, confounding may be an issue in estimating the causal effect of LMWH on the incidence of bleeding and thromboembolic events in these studies. Therefore, in this study, we estimated the risk of major bleedings, thromboembolic events, neurologic complications, and all-cause mortality in CF-LVAD patients, in an outpatient setting, during periods of bridging with LMWH for subtherapeutic INRs, using a design that mimics a randomized trial (quasiexperimental design).

## Methods

### Study Population

This cohort study included patients who received a CF-LVAD (HVAD [Medtronic, Framingham, MA] or HeartMate 3 [Abbott, Abbott Park, IL]) between January 1, 2010, and August 1, 2024, at the Leiden University Medical Center (LUMC), the Netherlands. HeartWare device and HeartMate 3 were the only two types of CF-LVAD implanted at the LUMC. All patients were 18 years or older, had end-stage HF, and were ineligible for heart transplantation at the moment of CF-LVAD implantation.

### Data Collection

Baseline patient characteristics, including demographic variables, etiology of HF, Interagency Registry for Mechanically Assisted Circulatory Support (INTERMACS) profiles, data concerning the LVAD implantation, comorbidities, medical history related to cardiovascular diseases and comedication related to HF and anticoagulation were collected from the electronic patient records in the LUMC. Data regarding anticoagulation management and INRs during follow-up were collected from the electronic outpatient records of the regional anticoagulation clinics. Patients were excluded when no INR data was available in case the patients were not discharged from the hospital due to death. In total, 118 patients received a CF-LVAD at the LUMC during the study period, of which 92 (78%) were discharged from the hospital and had INR data available. The study protocol was approved by the medical ethics committee of the LUMC.

### Bridging With Low-Molecular-Weight Heparin and International Normalized Ratio Measurements

All patients received VKA and dosage was adjusted based on an individual therapeutic INR range decided by the treating physician after the implantation. At home, patients were instructed to perform INR self-tests from point-of-care testing, at least twice a week or more frequently if necessary. Then, the dose of VKA was adjusted by anticoagulation clinic physicians from the regional anticoagulation clinics in Leiden. Patients were instructed to self-administer LMWH in a therapeutic dose whenever their INR was below their target range and to continue the double antithrombotic therapy using VKA and antiplatelet medication (clopidogrel 75 mg for HVAD patients and aspirin 80 for HeartMate 3 patients). Due to the absence of information on starting date of LMWH use in the electronic patient data of the anticoagulation clinic and information from the pharmacy that provided the LMWH, we assumed that patients used LMWH when their INR was subtherapeutic as instructed by their treating physician.

### Outcomes

Primary outcomes included major bleeding, thromboembolic events, neurologic complications, and all-cause mortality after CF-LVAD implantation. Thromboembolic events were defined according to INTERMACS criteria and included pump thrombosis, arterial (noncentral nervous system), and venous thromboembolism.^18^ Major bleedings were also classified according to the INTERMACS criteria and included bleedings that required red blood cell transfusion, bleedings that required surgical intervention and fatal bleedings (Types 3, 4, and 5, respectively).^18^ Neurologic complications included ischemic and hemorrhagic strokes and neurologic dysfunction without central nervous system injury (INTERMACS Types 1 and 3, respectively). All adverse events were adjudicated by two treating physicians.

### Statistical Analysis

Categorical variables were described as numbers (percentage) while continuous variables were described with median (interquartile range [IQR]). Patients were followed from the start of VKA after CF-LVAD implantation until study completion date (August 1, 2024) or death, whichever occurred first. In calculating the follow-up time until an outcome, we did not consider the occurrence of the other outcomes. For example, if a patient experienced both a thromboembolic event and a major bleeding, we calculated separate follow-up times for each analysis. Thus, all follow-up from the start of VKA to the major bleeding was included in the analysis of major bleeding, and all follow-up until the thromboembolic event was included in the thromboembolic events analysis.

A regression discontinuity (RD) design (a quasiexperimental design) was used to estimate the incidence of the primary outcomes during the LMWH and no LMWH treatment periods.^19^ An RD design can be used when the treatment is assigned based on the threshold of a continuous variable. In our case, patients initiated LMWH treatment when their INR was below the lower INR target range (*i.e.*, INR threshold for treatment). This means that patients with a subtherapeutic INR measurement (*i.e.*, below the lower INR target range) were treated with LMWH, whereas patients with a therapeutic INR were not. LMWH treatment was thus determined by INR, which is a continuous variable subject to random variability due to, for example, random error, sampling variability, and chance factors. This variability in INR ensures that patients close to the treatment threshold are randomly assigned to receive LMWH or not, mimicking the random treatment assignment of a randomized clinical trial and controlling for confounding.

We estimated incidence rates (IRs) per patient-year and hazard ratios (HR) with 95% confidence intervals (95% CI) of the primary outcomes by means of time-dependent Cox regression, comparing patients during periods of treatment with LMWH (with an INR just below the target range) with patients during periods without LMWH treatment (with an INR just above). Robust standard errors were used to account for individual clusters. This analysis was performed using five different predefined bandwidths around the lower INR target range, *i.e.*, ±0.1, ±0.2, ±0.3, ±0.4, and ±0.5 around the lower INR target range. For instance, if a patient had a lower INR target range of 2.0, INRs from 1.7 to 2.3 were included in the analysis with bandwidth ±0.3 with 1.7–2.0 as a LMWH period and 2.0–2.3 as a non-LMWH period. Moreover, the period until two subsequent therapeutic INR measurements after a subtherapeutic INR was excluded from the analyses, as patients were instructed to continue LMWH until two therapeutic INRs were achieved.

Statistical analyses were performed using R, version 4.2.1 (R Foundation for Statistical Computing, Vienna, Austria).

## Results

### Patient Characteristics

A total of 92 patients who underwent CF-LVAD implantation at the LUMC between January 1, 2010, and August 1, 2024, who were discharged home and had INR measurements available were included (Table 1). The patients had a median age at implantation of 63 years (IQR 59–69 years) and 73 (79%) were male. Moreover, 52 (57%) had ischemic HF, 43 (47%) had INTERMACS classification 3, and 88 (96%) had CF-LVAD implantation as destination therapy. The majority of patients (73, 79%) had an HVAD implanted, whereas 19 (21%) received a HeartMate 3. Patients were followed for a median of 24 months (IQR 11–49 months).

**Table 1.** Baseline Characteristics of CF-LVAD Patients

	n = 92
At CF-LVAD implantation	
Age (years), median (IQR)	63 (59–69)
Sex (male)	73 (79)
BMI (kg/m^2^), median (IQR)	25.5 (22.6–29.2)
Etiology heart failure	
Ischemic	52 (57)
Nonischemic	37 (40)
Congenital	3 (3)
INTERMACS classification	
1	3 (3)
2	13 (14)
3	43 (47)
4	20 (22)
5	13 (14)
Device strategy	
Bridge to decision	4 (4)
Destination therapy	88 (96)
Comorbidities prior CF-LVAD implantation	
Diabetes	22 (24)
Hypertension	29 (32)
Coronary artery disease	58 (63)
Atrial fibrillation	50 (56)
Neurologic events	
TIA	9 (11)
Ischemic stroke	12 (15)
Hemorrhagic stroke	3 (4)
Thromboembolic event*	12 (13)
Major bleeding*	20 (22)
Mechanical valve replacement	5 (5)
History of cancer	24 (26)
Medication prior CF-LVAD implantation	
β blocker	56 (61)
ACE inhibitor	10 (11)
Angiotensin receptor II blocker	7 (8)
Sacubitril/valsartan	7 (8)
Aldosterone antagonist	65 (71)
Loop diuretic	63 (68)
Thiazide diuretic	10 (11)
Amiodarone	42 (46)
Statin	51 (55)
Antiplatelets	11 (12)
Vitamin K antagonist	30 (33)
LMWH	36 (39)

ACE, angiotensin-converting enzyme; BMI, body mass index; CF-LVAD, continuous-flow left ventricular assist device; INTERMACS, Interagency Registry for Mechanically Assisted Circulatory Support; INR, international normalized ratio; IQR, interquartile range; LMWH, low-molecular-weight heparin; TIA, transient ischemic attack.

* Hemorrhagic stroke, ischemic stroke, and TIA excluded.

### Low-Molecular-Weight Heparin Bridging Episodes

The patients had a total of 30,990 INR measurements. Within the different analyses including INRs ±0.1, ±0.2, ±0.3, ±0.4, and ±0.5 around the lower INR target range, 1,108, 1,879, 2,430, 2,815, and 3,044 “bridging INRs” and 3,366, 5,778, 8,508, 11,453, and 14,119 therapeutic INRs were included, respectively, over a follow-up time of 143.6 patient-years. There was an average of 3 days between consecutive INR measurements (IQR 3–4 days).

### Major Bleeding

**Figure 1.**
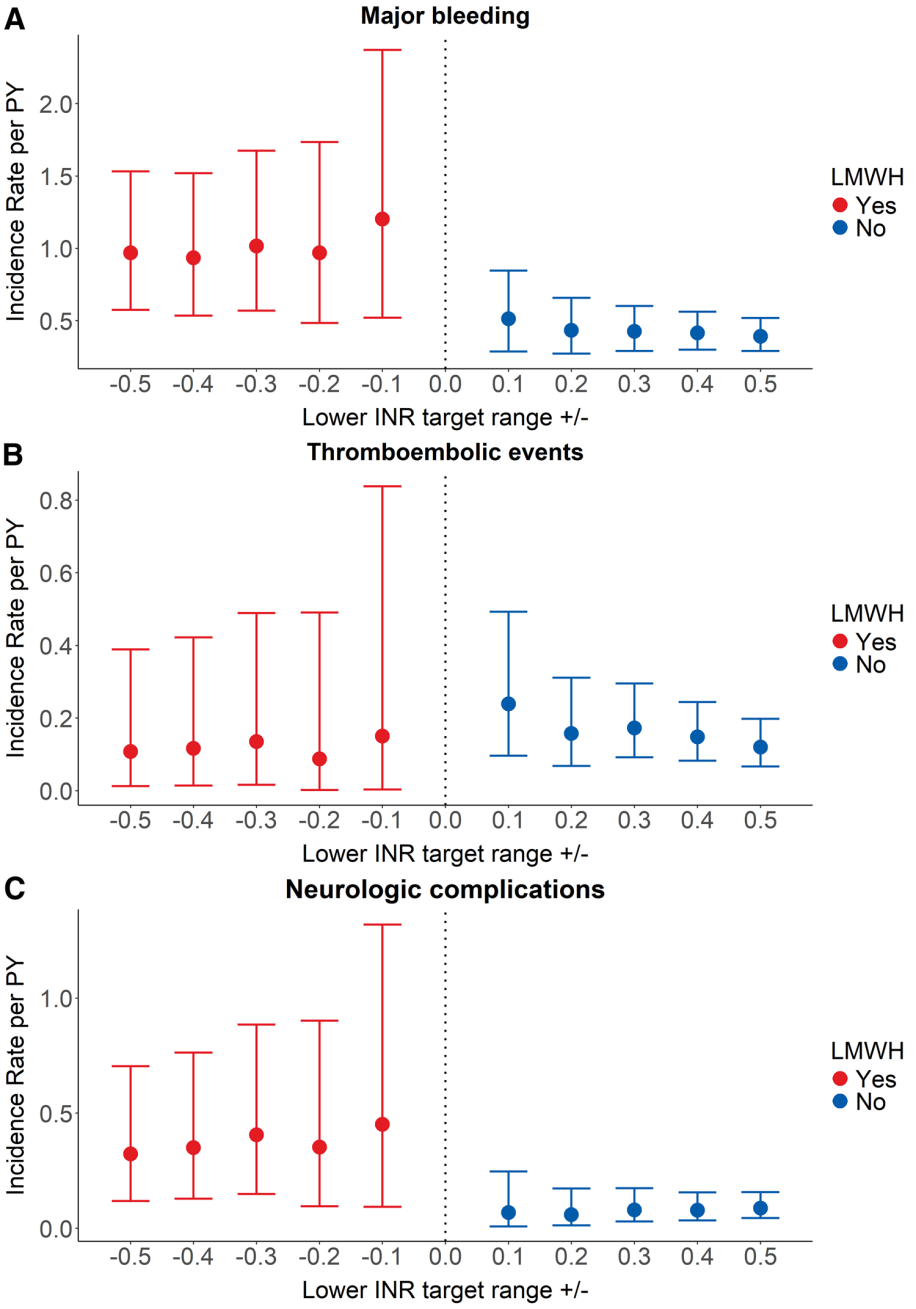
Incidence rates and 95% confidence intervals of major bleeding (**A**), thromboembolic events (**B**), and neurologic complications (**C**) during LMWH treatment (red) and no LMWH (blue). INR, international normalized ratio; LMWH, low-molecular-weight heparin; PY, patient-year.

**Figure 2.**
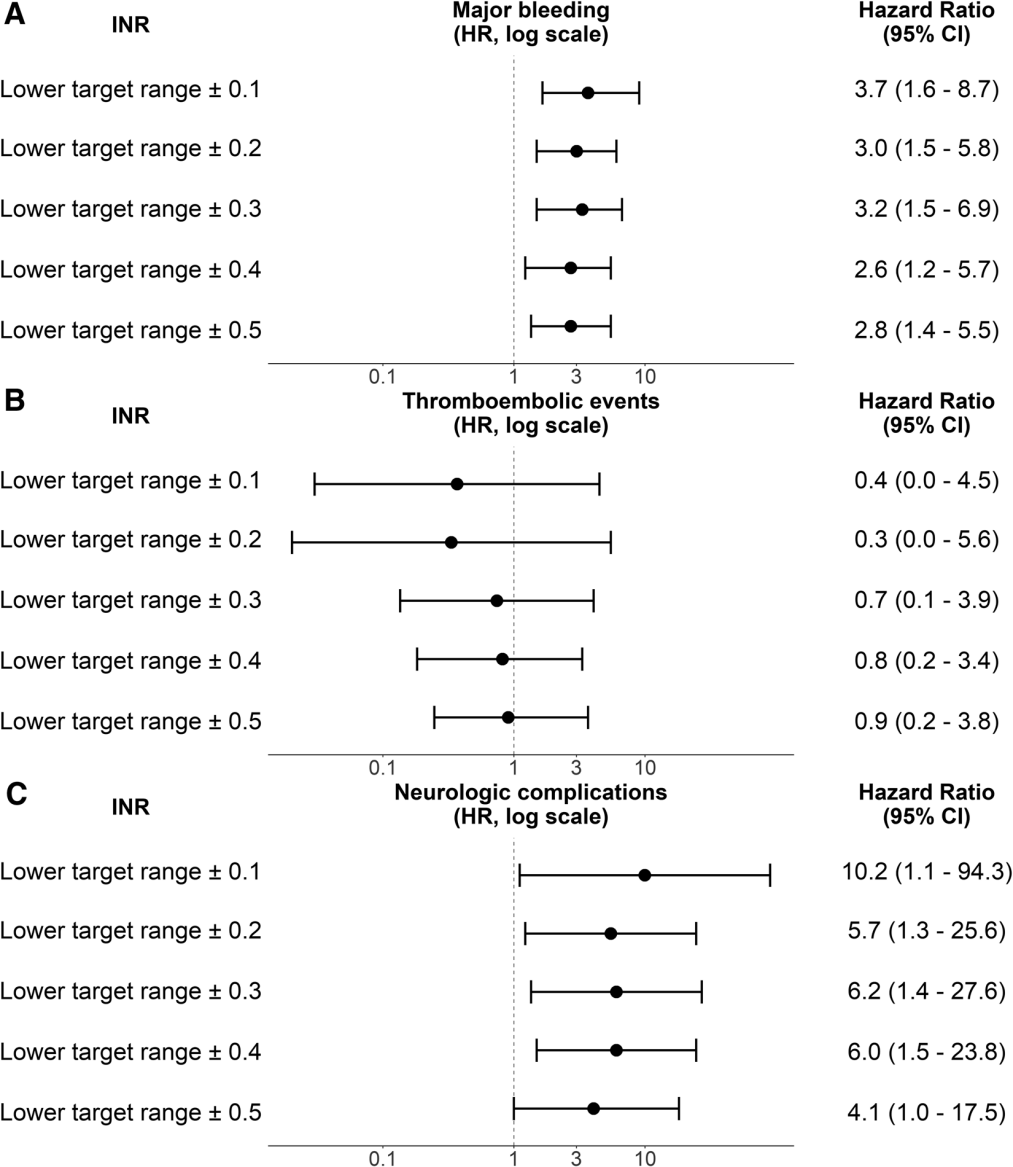
Hazard ratios and 95% CIs of major bleeding (**A**), thromboembolic events (**B**), and neurologic complications (**C**) during LMWH treatment *vs.* no LMWH. In the graph, HRs are depicted on the logarithmic scale. CI, confidence interval; HR, hazard ratio; INR, international normalized ratio; LMWH, low-molecular-weight heparin.

A total of 67 major bleedings were documented during the included INR measurement periods (Supplementary Table 1, Supplemental Digital Content, http://links.lww.com/ASAIO/B486). Almost all bleeding (65) required red blood cell transfusion. In all analyses, IRs of major bleeding per patient-year were higher during periods of LMWH treatment compared with periods of no LMWH (Figure 1). In the analysis including INRs ±0.5, 18 major bleeding occurred during 18.6 patient-years of follow-up in periods of bridging with LMWH and 49 major bleeding during 125.0 patient-years of follow-up in periods of no LMWH. This resulted in a higher IR of major bleeding during bridging with LMWH compared with no bridging, with an IR ratio of 2.5 (95% CI, 1.4–4.2) (Figure 1 and Supplementary Table 2, Supplemental Digital Content, http://links.lww.com/ASAIO/B486). In all other analyses, except those including INRs ±0.1, the risk of major bleeding was threefold increased during LMWH compared with no LMWH (Figure 2). In the analysis including INRs ±0.1, bridging with LMWH was associated with a 3.7-fold (95% CI, 1.6–8.7) increased risk of major bleeding compared with periods of no bridging.

### Thromboembolic Events

Overall, thrombotic events during the included INR measurements were rare, as a total of 17 events were included, of which 16 were pump thrombosis (Supplementary Table 1, Supplemental Digital Content, http://links.lww.com/ASAIO/B486). Only eight thrombotic events occurred during INRs ±0.1 and ±0.2 (Figure 1 and Supplementary Table 2, Supplemental Digital Content, http://links.lww.com/ASAIO/B486). Treatment with LMWH showed similar IRs of thromboembolic events compared with untreated periods in all analyses. Overall, LMWH treatment was associated with a similar risk of thromboembolic events as no LMWH (HR of 0.9 [95% CI, 0.2–3.8] when including INRs ±0.5, Figure 2).

### Neurologic Complications

Neurologic complications occurred in 15 patients during the included periods of INR measurements. The most common neurologic event was ischemic stroke or a neurologic dysfunction without central nervous system injury (15 events), while only two patients suffered from a hemorrhagic stroke (Supplementary Table 1, Supplemental Digital Content, http://links.lww.com/ASAIO/B486). Rates of neurologic complications were higher during periods of LMWH treatment in all analyses, compared with no LMWH (Figure 1 and Supplementary Table 2, Supplemental Digital Content, http://links.lww.com/ASAIO/B486). In the analysis including INRs ±0.5, patients had a 4.1-fold (95% CI, 1.0–17.5) increased risk of neurologic complications during periods of LMWH compared with no LMWH (Figure 2). This risk was 10.2-fold (95% CI, 1.1–94.3) increased during bridging compared with no bridging in the analysis including INRs ±0.1.

### All-Cause Mortality

**Figure 3.**
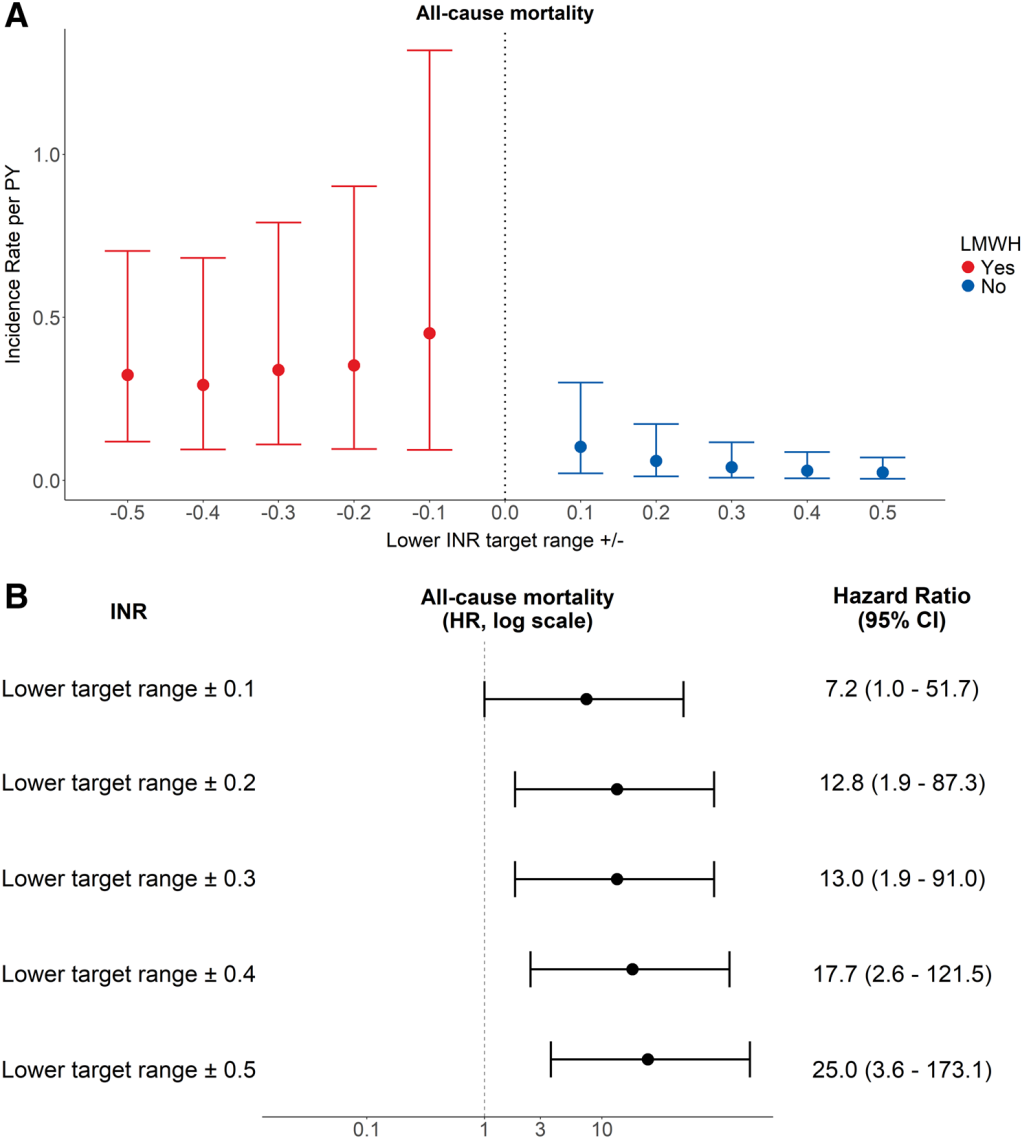
Incidence rates (**A**) and HRs (**B**) (with 95% CIs) of all-cause mortality during LMWH treatment *vs.* no LMWH. In the graph, HRs are depicted on the logarithmic scale. CI, confidence interval; HR, hazard ratio; INR, international normalized ratio; LMWH, low-molecular-weight heparin; PY, patient-year.

Sixty patients (65%) died during follow-up, of which nine patients died during a period between INR measurements ±0.5 (Supplementary Table 1, Supplemental Digital Content, http://links.lww.com/ASAIO/B486). Higher IRs of all-cause mortality were found during LMWH treatment compared with no LMWH treatment in all analyses (Figure 3 and Supplementary Table 2, Supplemental Digital Content, http://links.lww.com/ASAIO/B486). The analysis including INRs ±0.5 showed a 25.0-fold (95% CI, 3.6–173.1) increased risk of mortality in the patients treated with LMWH compared with the untreated, and the risk was similarly increased in the other analyses (Figure 3 and Supplementary Table 2, Supplemental Digital Content, http://links.lww.com/ASAIO/B486).

## Discussion

We aimed to investigate the risk of adverse events, in terms of major bleedings, thromboembolic events, and all-cause mortality, in CF-LVAD patients during periods of bridging with LMWH during subtherapeutic INRs *versus* no bridging. Our main results showed that bridging therapy was associated with a threefold increased risk of major bleeding compared with no bridging. This risk was almost fourfold in the analysis considering INR ±0.1 to the lower target range. Thromboembolic events were infrequent in our analysis and occurred to a similar extent during bridging and no bridging. Also, neurologic complications were not frequent but occurred more frequently during LMWH use. Additionally, we observed an increased risk of all-cause mortality during bridging *versus* no bridging. However, this finding should be interpreted with caution, as only nine patients died during the included INRs and therefore the CIs are wide.

Balancing the risk of bleeding and thrombosis remains the most challenging task in the management of long-term anticoagulation in patients with a CF-LVAD. The high incidence of thromboembolic complications warrants the use of double antithrombotic therapy.^11,20^ The thromboembolic risk, in particular the risk of pump thrombosis, differs also per type and generation of device, with the HeartMate 3 showing the lower rates of thromboembolic events.^21^ Maintaining the INR strictly in the therapeutic range is recommended by current guidelines,^14^ however the time spent in the therapeutic range is lower in CF-LVAD patients compared with other VKA-treated populations.^22^ As expected, an increased incidence of adverse outcomes was observed when the control of anticoagulation is suboptimal compared with good anticoagulation control.^23^ Moreover, the incidence of thrombotic events was inversely correlated with INR levels, with the highest rate of events observed when the INR is below 1.5.^24^ Therefore, bridging with LMWH during periods of subtherapeutic anticoagulation is suggested by the current guidelines.^14^ However, the evidence available on the efficacy and safety of bridging is limited to a few observational studies with conflicting results and no randomized trial has ever been conducted.

Previously, Bhatia *et al.*^7^ reported a fourfold increased incidence of major bleeding and no difference in the incidence of thromboembolic events during periods of bridging compared with nonbridged periods, which is in line with our findings. In another study, Rabon *et al.*^15^ found that bridging with LMWH or unfractionated heparin increased the risk of major bleeding (HR, 2.3; 95% CI, 1.0–4.9 for LMWH) while the risk of thromboembolic was not decreased (adjusted HR, 1.6; 95% CI, 0.3–8.7). It must be noted, however, that these results might be biased by confounding by indication, as patients were bridged at the discretion of the treating physician.^15^ In contrast, two other studies found no difference in the rates of major bleeding and thromboembolic events during bridging.^16,17^ In both studies, the rates of major bleeding and thromboembolic events were overall low. These different results might be explained by a short duration of bridging (median of 6^16^ and 2 days,^17^ respectively), shorter follow-up, and by different baseline thrombotic and bleeding risks of the included population compared with our study.^17^

Our results suggest that bridging with LMWH does not offer a benefit of preventing thromboembolic complications and instead increases the risk of major bleeding. In other populations, such as patients with atrial fibrillation or patients with a mechanical heart valve, bridging for a subtherapeutic INR in outpatient settings is not recommended, as the absolute risk of a thrombotic event is low.^25,26^ A strategy to reduce the risk of bleeding during bridging may be to lower the INR threshold for bridging or to reduce the dose of LMWH. A recent pilot study, in which patients were not bridged unless their INR was <1.8, reported no difference in clinical outcomes and the anticoagulant effect (measured by thromboelastography) between patients treated with a reduced LMWH dose and patients treated with full dose.^27^ However, a previous article indicated that the incidence of thromboembolic events was high also for INRs between 1.5 and 1.9.^24^ This could be different for the new generation device such as HeartMate 3, in which the risk of thrombosis appears to be lower.^21^ We could not stratify our results for type of device, as only 19 patients received a HeartMate 3 in our population. Nevertheless, as the absolute risk of pump thrombosis seems lower with HeartMate 3, the risk of major bleeding associated with bridging becomes even more relevant when assessing the balance between the risks and benefits of bridging.

Several important aspects should be considered in our study. We included all CF-LVAD patients implanted in our center with no loss to follow-up, which resulted in an unselected population with more than 10 years of follow-up. In addition, we used a RD design, which is a quasiexperimental method that mimics a randomized trial.^19^ Previous studies have already demonstrated that this approach yields similar results to randomized trials.^28^ We performed the analysis multiple times, using different bandwidths of the INR around lower INR target range. This approach allows us to balance the trade-off between more power when using a larger bandwidth (thus including a larger number of events during a broader range of subtherapeutic INRs), and a more comparable group, when using a smaller bandwidth. This design solves the issue of confounding that affected previous observational studies.^19^ However, an RD by design estimates the causal effect around the treatment threshold, therefore our results are not applicable to patients with very low INRs and only apply to patients with a subtherapeutic INR which is less than 0.5 of their lower INR target range. Nonetheless, we believe that this is the population in which the benefits and the risks of bridging should be weighted the most. In addition, our population is mainly composed of patients who received an HVAD. Moreover, we included only outpatient measures of INRs, which makes our results applicable to the outpatient setting. We did not have information on the exact date of LMWH administration and assumed that the patients used LMWH in case of a subtherapeutic INR. However, this potential misclassification could only have led to an underestimation of the risk of bleeding and thrombosis during bridging. Finally, our cohort was relatively older than those included in previous studies and composed of patients implanted as destination therapy,^7,15,17^ therefore our results are only generalizable to similar populations.

In conclusion, bridging therapy with LMWH for subtherapeutic INR in CF-LVAD patients was associated with a threefold increased risk of major bleeding and a fourfold increased risk of neurologic complications, which were mostly ischemic strokes. Thrombotic events were infrequent and, although the CIs were wide, there was an increased risk of all-cause mortality during bridging. Therefore, bridging for a subtherapeutic INR in CF-LVAD patients should be carefully considered.

## Supplementary Material
