# Supplementary material for: Bridging Therapy and Risk of Bleeding and Thrombosis in Continuous-Flow Left Ventricular Assist Device Patients: A Quasi-Experimental Study
Source: ASAIO J. 2025 Apr 29;71(12):964–71. doi: 10.1097/MAT.0000000000002447 (PMC12646858; doi:10.1097/MAT.0000000000002447)
Supplement: Supplementary file 1 [file mat-71-0964-s001.pdf]

Bridging therapy and risk of bleeding and thrombosis in continuous-flow left ventricular assist device patients: a quasi-experimental study

Supplementary Material

Supplementary Table 1. Adverse events included in the analyses

	events (patients)
**Major bleeding events**	67 (27)
Requiring red blood cell transfusion	65 (26)
Requiring surgical intervention	1 (1)
Probable fatal bleeding	1 (1)
**Thromboembolic events**	17 (9)
Device thrombus	16 (8)
Major device malfunction resulting in hospitalization	1 (1)
**Neurologic complications**	17 (15)
Ischemic stroke	9 (8)
Neurologic dysfunction without central nervous system injury	6 (7)
Hemorrhagic stroke	2 (2)
**Deaths**	9 (9)

Supplementary Table 2. Incidence rates and incidence rate ratios of adverse events during periods of INR below the treatment threshold (treated with LMWH) and just above (no LMWH).

Adverse event		Events (n)	Person-time (years)	Incidence rate (per PY; 95% CI)	Incidence rate ratio (95% CI)
**Major bleeding**					
**Lower INR target range ± 0.1**	No LMWH	15	29.3	0.6 (0.3 – 0.8)	Ref.
	LMWH	8	6.6	1.2 (0.5 – 2.4)	2.3 (1.0 – 5.5)
**Lower INR target range ± 0.2**	No LMWH	22	50.7	0.4 (0.3 – 0.7)	Ref.
	LMWH	11	11.4	1.0 (0.5 – 1.7)	2.2 (1.1 – 4.6)
**Lower INR target range ± 0.3**	No LMWH	32	75.2	0.4 (0.3 – 0.6)	Ref.
	LMWH	15	14.8	1.0 (0.6 – 1.7)	2.4 (1.3– 4.4)
**Lower INR target range ± 0.4**	No LMWH	42	101.2	0.4 (0.3 – 0.6)	Ref.
	LMWH	16	17.1	0.9 (0.5 – 1.5)	2.2 (1.3 – 4.0)
**Lower INR target range ± 0.5**	No LMWH	49	125.0	0.4 (0.3 – 0.5)	Ref.
	LMWH	18	18.6	1.0 (0.6 – 1.5)	2.5 (1.4 – 4.2)
**Thromboembolic events**					
**Lower INR target range ± 0.1**	No LMWH	7	29.3	0.2 (0.1 – 0.5)	Ref.
	LMWH	1	6.6	0.2 (0.004 – 0.8)	0.6 (0.1 – 5.1)
**Lower INR target range ± 0.2**	No LMWH	8	50.7	0.2 (0.07 – 0.3)	Ref.
	LMWH	1	11.4	0.1 (0.002 – 0.5)	0.6 (0.1 – 4.5)
**Lower INR target range ± 0.3**	No LMWH	13	75.2	0.2 (0.1 – 0.3)	Ref.
	LMWH	2	14.8	0.1 (0.02 – 0.5)	0.8 (0.1 – 3.5)
**Lower INR target range ± 0.4**	No LMWH	15	101.2	0.1 (0.1 – 0.2)	Ref.
	LMWH	2	17.1	0.1 (0.01 – 0.4)	0.8 (0.2 – 3.4)
**Lower INR target range ± 0.5**	No LMWH	15	125.0	0.1 (0.1 – 0.2)	Ref.
	LMWH	2	18.6	0.1 (0.01 – 0.4)	0.9 (0.2 – 3.9)
**Neurologic complications**					
**Lower INR target range ± 0.1**	No LMWH	2	29.3	0.1 (0.01 – 0.2)	Ref.
	LMWH	3	6.6	0.5 (0.1 – 1.3)	6.6 (1.1 – 39.5)
**Lower INR target range ± 0.2**	No LMWH	3	50.7	0.1 (0.01 – 0.2)	Ref.
	LMWH	4	11.4	0.4 (0.1 – 0.9)	6.0 (1.3 – 26.6)
**Lower INR target range ± 0.3**	No LMWH	6	75.2	0.1 (0.03 – 0.2)	Ref.
	LMWH	6	14.8	0.4 (0.2 – 0.9)	5.1 (1.6 – 15.8)
**Lower INR target range ± 0.4**	No LMWH	8	101.2	0.1 (0.03 – 0.2)	Ref.
	LMWH	6	17.1	0.4 (0.1 – 0.8)	4.4 (1.5 – 12.7)
**Lower INR target range ± 0.5**	No LMWH	11	125.0	0.1 (0.04 – 0.2)	Ref.
	LMWH	6	18.6	0.3 (0.1 – 0.7)	3.7 (1.3 – 9.9)
**All-cause mortality**					
**Lower INR target range ± 0.1**	No LMWH	3	29.3	0.1 (0.02 – 0.3)	Ref.
	LMWH	3	6.6	0.5 (0.1 – 1.3)	4.4 (0.9 – 21.8)
**Lower INR target range ± 0.2**	No LMWH	3	50.7	0.1 (0.01 – 0.2)	Ref.
	LMWH	4	11.4	0.4 (0.1 – 0.9)	6.0 (1.3 – 26.6)
**Lower INR target range ± 0.3**	No LMWH	3	75.2	0.04 (0.01 – 0.1)	Ref.
	LMWH	5	14.8	0.3 (0.1 – 0.8)	8.5 (2.0 – 35.5)
**Lower INR target range ± 0.4**	No LMWH	3	101.2	0.03 (0.01 – 0.1)	Ref.
	LMWH	5	17.1	0.3 (0.1 – 0.7)	9.9 (2.4 – 41.2)
**Lower INR target range ± 0.5**	No LMWH	3	125.0	0.02 (0.005 – 0.1)	Ref.
	LMWH	6	18.6	0.3 (0.1 – 0.7)	13.5 (3.4 – 53.8)

The analysis “Lower INR target range ± 0.1” included events during a period of time the patients had INRs between the lower value of the target range -0.1 and the lower value + 0.1. For example, if a patient had an INR lower value of the target range value of 2.0, events during INRs from 1.9 to 2.1 were included in this analysis.
PY, patient-years; CI, confidence interval; INR, international normalized ratio; LMWH, low molecular weight heparin.
